# Late Morbidity and Mortality in Survivors of Childhood Ependymoma: A Report from the Childhood Cancer Survivor Study (CCSS)

**DOI:** 10.3390/cancers17223669

**Published:** 2025-11-15

**Authors:** Katharine R. Lange, Peter de Blank, Mengqi Xing, Sedigheh Mirzaei, Deo Kumar Srivastava, Kevin Oeffinger, Joseph Neglia, Kevin Krull, Paul C. Nathan, Rebecca Howell, Kirsten K. Ness, Lucie M. Turcotte, Wendy Leisenring, Gregory T. Armstrong, Tara Brinkman, Daniel C. Bowers, Mehmet Fatih Okcu

**Affiliations:** 1 Hackensack Meridian School of Medicine, Nutley, NJ 07110, USA; 2 Department of Pediatrics, Hackensack University Medical Center, Hackensack, NJ 07601, USA; 3 University of Cincinnati College of Medicine, Cincinnati, OH 45229, USA; peter.deblank@cchmc.org; 4 Cincinnati Children’s Hospital Medical Center, Cincinnati, OH 45229, USA; 5 St. Jude Children’s Research Hospital, Memphis, TN 38105, USA; mengqi.xing@stjude.org (M.X.); sadie.mirzaei@stjude.org (S.M.); kumar.srivastava@stjude.org (D.K.S.); kevin.krull@stjude.org (K.K.); kiri.ness@stjude.org (K.K.N.); greg.armstrong@stjude.org (G.T.A.); tara.brinkman@stjude.org (T.B.); 6 Department of Medicine, Duke University, Durham, NC 27705, USA; kevin.oeffinger@duke.edu; 7 Department of Pediatrics, University of Minnesota, Minneapolis, MN 55454, USA; jneglia@umn.edu (J.N.); turc0023@umn.edu (L.M.T.); 8 The Hospital for Sick Children, Toronto, ON M5G 1E8, Canada; paul.nathan@sickkids.ca; 9 Department of Radiation Physics, The University of Texas at MD Anderson Cancer Center, Houston, TX 77030, USA; rhowell@mdanderson.org; 10 Fred Hutchinson Cancer Research Center, Seattle, WA 98109, USA; wleisenr@fredhutch.org; 11 Department of Pediatrics, University of Texas Southwestern Medical Center, Dallas, TX 75235, USA; daniel.bowers@utsouthwestern.edu; 12 Texas Children’s Hospital, Baylor College of Medicine, Houston, TX 77030, USA; mfokcu@texaschildrens.org

**Keywords:** pediatric ependymoma, late morbidity and mortality, radiation and chemotherapy

## Abstract

Background/Objectives: Treatment of childhood ependymoma evolved from 1970 to 1999 by reducing radiation volumes and incorporating chemotherapy. The impact of these changes on long-term health outcomes remains unknown. In this report, we evaluated temporal changes in all-cause and cause-specific late mortality, chronic health conditions (CHCs), and subsequent neoplasms (SNs) in the Childhood Cancer Survivor Study (CCSS) cohort of adult survivors of pediatric ependymoma, diagnosed between 1970 and 1999. Methods: A total of 404 five-year survivors of ependymoma (47.5% female, 80.7% non-Hispanic White, median 6 (range 0–20) years at diagnosis, 22 (5–49) years from diagnosis) diagnosed between 1970 and 1999 and enrolled in the Childhood Cancer Survivor Study were evaluated for late (>5 years from diagnosis) mortality, SNs, and CHCs. Outcomes were analyzed by diagnosis decade, radiotherapy, and chemotherapy exposure. Gray’s test compared cumulative incidences. Multivariable piecewise exponential models estimated relative risks (RRs). Results: Whole-brain radiation exposure decreased over time (42.9% (1970s) to 2.7% (1990s)), while focal radiation (21.4% to 68.9%), and chemotherapy (29.5% to 50.2%) use increased. Fifteen-year all-cause late mortality (incidence, 95% CI) remained similar across decades: 1970s (9.3%, 3.4–18.8%), 1980s (14.7%, 9.4–21.2%), 1990s (10.3%, 6.7–14.9%). All-cause late mortality was higher after treatment with whole-brain radiation (22.5%, 11.2–36.5%) compared to focal radiation (11.4%, 7.5–16.1%) or no brain radiation (3.5%, 0.9–9.1%) (*p* < 0.001), and with chemotherapy (14.4%, 9.6–20.0%) versus without (6.8%, 3.8–11.0%) (*p* = 0.004). Compared to no brain radiation, the RR (95% CI) of grade 3–4 CHCs increased among survivors treated with focal (2.6, 1.3–5.4) and whole-brain radiation (3.5, 1.5–8.1), while chemotherapy was not associated with CHCs or SNs. Conclusions: Despite reduced radiation volumes and increased use of chemotherapy, late mortality and morbidity among pediatric ependymoma survivors remained largely unchanged across treatment decades.

## Simple Summary

This study provides the most comprehensive evaluation to date of long term outcomes among five year survivors of pediatric ependymoma. Between 1970 and 1999, treatment for pediatric ependymoma evolved to reduce cranial radiation volumes and incorporate chemotherapy for some patients. But, late morbidity and mortality have not improved among pediatric ependymoma survivors despite these treatment changes. Reduced cranial radiation volume was associated with a reduced risk for late mortality and decreased grade 3–4 chronic health conditions. Our findings reveal that rates of late mortality and serious chronic health conditions have not substantially improved over the decades studied though reduced whole-brain radiation exposure is associated with lower late mortality and morbidity, supporting efforts to minimize radiation fields when safe and feasible.

## 1. Introduction

Pediatric ependymoma accounts for approximately 190 new cases annually in the United States, representing 5.5% of childhood brain tumors [1], and the underlying causes for this rare disease are largely unknown. Overall survival for most pediatric cancers has improved over the decades, and as many as 70% of newly diagnosed pediatric ependymoma patients in the current era will become five-year survivors [1]. However, late onset mortality and morbidity, occurring after five years from diagnosis, and the effect of recent advances in treatment on ependymoma survivors are poorly understood.

Contemporary therapy for pediatric ependymoma consists of maximal surgical resection followed by radiation therapy. Although some ependymomas can be cured without radiation exposure, most require radiation to achieve a lasting cure [2]. Radiation therapy is often accepted as a standard of care because of its importance in achieving a cure, despite its effects on cognitive development and long-term health outcomes [3,4,5]. Over the past decades, radiation therapy for non-metastatic ependymoma has moved from whole-brain irradiation, often including spinal irradiation, to more focal therapy [6]. Current radiation therapies do not work in all cases and various chemotherapy regimens have attempted to augment or replace radiation therapy in children with ependymoma, but few have demonstrated prolonged survival [7,8,9].

Due to their tumor location and treatment, survivors of childhood brain tumors are at high risk for developing late morbidity or mortality. Common morbidities following ependymoma treatment include radiation-related subsequent neoplasms (SNs) (such as meningioma and high-grade glioma) and chronic health conditions (CHCs), especially endocrine and neurologic conditions, which may increase in cumulative incidence across the lifespan [7,10,11]. Younger age at diagnosis and treatment, as well as radiation dose, have been associated with increased risk of neurologic late effects [12,13].

In this report, we evaluated temporal changes in all-cause and cause-specific late mortality, CHCs, and SNs in the Childhood Cancer Survivor Study (CCSS) cohort of adult survivors of pediatric ependymoma, diagnosed between 1970 and 1999. This time period was chosen to allow for mature follow up data and to understand the effect of treatment changes on survivorship care for adult survivors of childhood ependymoma. Outcomes were compared by treatment era and by historical changes in treatment modality across three decades. Prior studies of late mortality and morbidity among pediatric cancer survivors enrolled in the CCSS have demonstrated continued improvements in outcomes over time that have been attributed to changes in therapy: specifically, reductions in therapeutic exposure for low-risk populations, technical advancements in surgery and radiation, and increased access to survivorship care [14,15,16].

## 2. Materials and Methods

The CCSS is a National Cancer Institute (NCI)-funded retrospective cohort with a longitudinal follow-up of five-year survivors of childhood cancer diagnosed between 1970 and 1999. Participants were treated at 31 participating institutions in the United States and Canada and were younger than 21 years at diagnosis. The study methods and design have been described in previous publications [17,18]. The institutional review boards at each of the centers participating in the CCSS approved the study, and participants or guardians provided informed consent.

The ICD-O diagnosis codes for ependymoma as well as distribution per era included in the cohort are listed in Table 1. Demographic and treatment characteristics were reported for three treatment eras: the 1970s, 1980s, and 1990s. Treatment variables included surgery, chemotherapy, and brain radiation. Treatment variables were only considered if they occurred within 5 years after diagnosis. Chemotherapy exposure was defined as yes or no. Table S1 shows chemotherapy drug exposure by era. We chose radiation treatment to the brain as an exposure variable due to the known late effects on childhood brain tumor survivors, though in this analysis, many patients who were categorized as receiving whole-brain radiation had cranio-spinal irradiation. Brain radiation was characterized as focal or whole-brain, based on radiotherapy record abstraction. For each individual, the maximum target dose to four previously defined brain regions [19], the frontal cortex, temporal lobe, posterior fossa, and parietal and occipital cortex were determined by summing the delivered doses from all overlapping treatment fields with irradiation to more than 50% of each region [20]. Treatments were defined as whole-brain radiation when all four regions were irradiated and the dose to each region was greater than or equal to 40Gy [19]. Focal brain radiation was defined as greater than or equal to 40Gy for at least one but not all four brain regions. Radiation to the spine was not considered in this analysis. Surgery was defined as any surgery, yes or no.

### 2.1. Mortality

Data regarding late mortality were obtained from the National Death Index, including cause of death according to the International Classification of Diseases, Ninth and Tenth Revisions (ICD-9 and ICD-10). Late mortality was measured from the cohort entry to either death or 31 December 2017, the last date of the National Death Index search. For deaths predating the National Death Index (1975–1979), death certificates were obtained. Deaths were categorized into three mutually exclusive groups: (1) ependymoma recurrence/progression, (2) external causes (accidents, injuries, trauma, suicide), or (3) other health-related causes (including subsequent neoplasms [SNs], cardiac, pulmonary, and all other causes). No cause of death was available for 11 survivors, including Canadian survivors, so they were excluded from analyses involving cause of death.

### 2.2. Chronic Health Conditions (CHCs)

A comprehensive set of questionnaires administered at baseline and up to four subsequent follow-up assessments were used by participants to report on various health conditions and their age at first diagnosis. Chronic health conditions (CHCs) were classified according to a modified framework based on the Common Terminology Criteria for Adverse Events (CTCAE), version 4.03, based on self or proxy reports. CHCs were categorized as mild (grade 1), moderate (grade 2), severe or disabling (grade 3), life threatening (grade 4), or fatal (grade 5) [21]. A panel of physician experts reviewed all reported conditions and determined severity grades [22]. The questionnaires captured only the first reported occurrence of each condition, except for subsequent neoplasms, which were documented separately. The follow-up for CHCs continued until the earliest occurrence of a CHC (or second CHC in multiple CHC analyses), late recurrence, subsequent neoplasm [SN], death, or the last questionnaire.

### 2.3. Subsequent Neoplasms (SNs)

SNs were defined as new neoplasms occurring five or more years from diagnosis. They were classified into two mutually exclusive categories: (1) subsequent malignant neoplasms (SMNs), consisting of invasive neoplasms with an International Classification of Diseases for Oncology (ICD-O, 3rd version) behavior code of 3, excluding nonmelanoma skin cancers, or (2) non-invasive neoplasms, including nonmelanoma skin cancers and benign meningiomas. When a pathology report was unavailable, the diagnosis was confirmed by death certificate, other medical records, or both [23]. SN/SMN were tracked until the date of diagnosis of SN/SMN, death, or the last questionnaire.

### 2.4. Statistical Methods

Demographic and treatment characteristics of survivors were described and compared across treatment eras. Continuous variables were analyzed using the Kruskal–Wallis test and categorical variables were assessed using Chi-square or Fisher exact tests.

Cumulative incidence of late mortality, CHCs, SNs, and SMNs was estimated 15 years from diagnosis, omitting events that occurred before cohort entry (up to 5 years after diagnosis). Gray’s test was used to compare cumulative incidence across treatment groups.

Piecewise exponential models were used to assess associations between chemotherapy and radiation exposures with late mortality, CHCs, SNs, and SMNs among survivors. Models were adjusted for attained age, sex, race, and ethnicity; for late mortality analyses, early recurrence and the ependymoma subtype were additionally included as covariates. Statistical analysis was completed using SAS v.9.4. A two-sided *p*-value of <0.05 was considered statistically significant.

## 3. Results

Among 25,665 CCSS participants, there were 404 five-year survivors of pediatric ependymoma. Ependymoma survivors were 47.5% female and 80.7% non-Hispanic White. The median age at diagnosis was 6 years (range 0–20 years), and survivors were followed for a median of 22 years (5–49 years) from diagnosis to last follow-up. Descriptive characteristics of the study population are shown in Table 1. Among survivors with known treatment information, 47.7% of patients were exposed to chemotherapy, 100% had surgery, and 75.7% were exposed to cranial radiation. Across the three decades, whole-brain radiation exposure decreased from 42.9% in the 1970s to 15.2% in the 1980s and 2.7% in the 1990s, while focal brain radiation exposure increased from 21.4% in the 1970s to 68.8% in the 1980s and 68.9% in the 1990s. Chemotherapy exposure increased from 29.5% in the 1970s to 50% in the 1980s and 50.2% in the 1990s. Across the three decades, the median age at diagnosis decreased from 10 years in the 1970s to 6 years in the 1980s and 5 years in the 1990s (*p* < 0.001). While the proportion of anaplastic ependymoma increased over the decades from 3.6% in the 1970s to 7.4% in the 1980s and 11.3% in the 1990s (*p* = 0.153), the distribution of ependymoma histopathological subtypes was not significantly different across the decades. Early recurrence within 5 years (*n* = 39) did not differ by treatment era (*p* = 0.25). Age at radiation did not differ across the decades.

Characteristics that might predict more aggressive disease or treatment choice were also investigated among survivors by treatment exposure, Table S2. Survivors exposed to chemotherapy were significantly younger at diagnosis compared to those not exposed (3 years (range 0–20 years) vs. 9 years (0–20 years), *p* < 0.0001). Compared to those not treated with chemotherapy, chemotherapy-exposed survivors were also more likely to be diagnosed with anaplastic ependymoma (*p* < 0.0001), be exposed to radiation (*p* < 0.0001), and have a recurrence within 5 years (*p* = 0.05). On the other hand, survivors treated with focal radiation were younger than survivors treated with whole-brain radiation (4 years (range 0–20 years) vs. 7.5 years (1–19 years), *p* = 0.017). Among all three radiation categories, survivors exposed to focal radiation were also more likely to be diagnosed with anaplastic ependymoma (14.6% compared to 5% (whole-brain radiation) and 0% (no brain radiation), *p* < 0.0001) and more likely to be exposed to chemotherapy (55.5% compared to 50% (whole-brain) and 27.4% (no brain radiation), *p* < 0.0001), (Table 1).

### 3.1. Late Mortality

**Figure 1:**
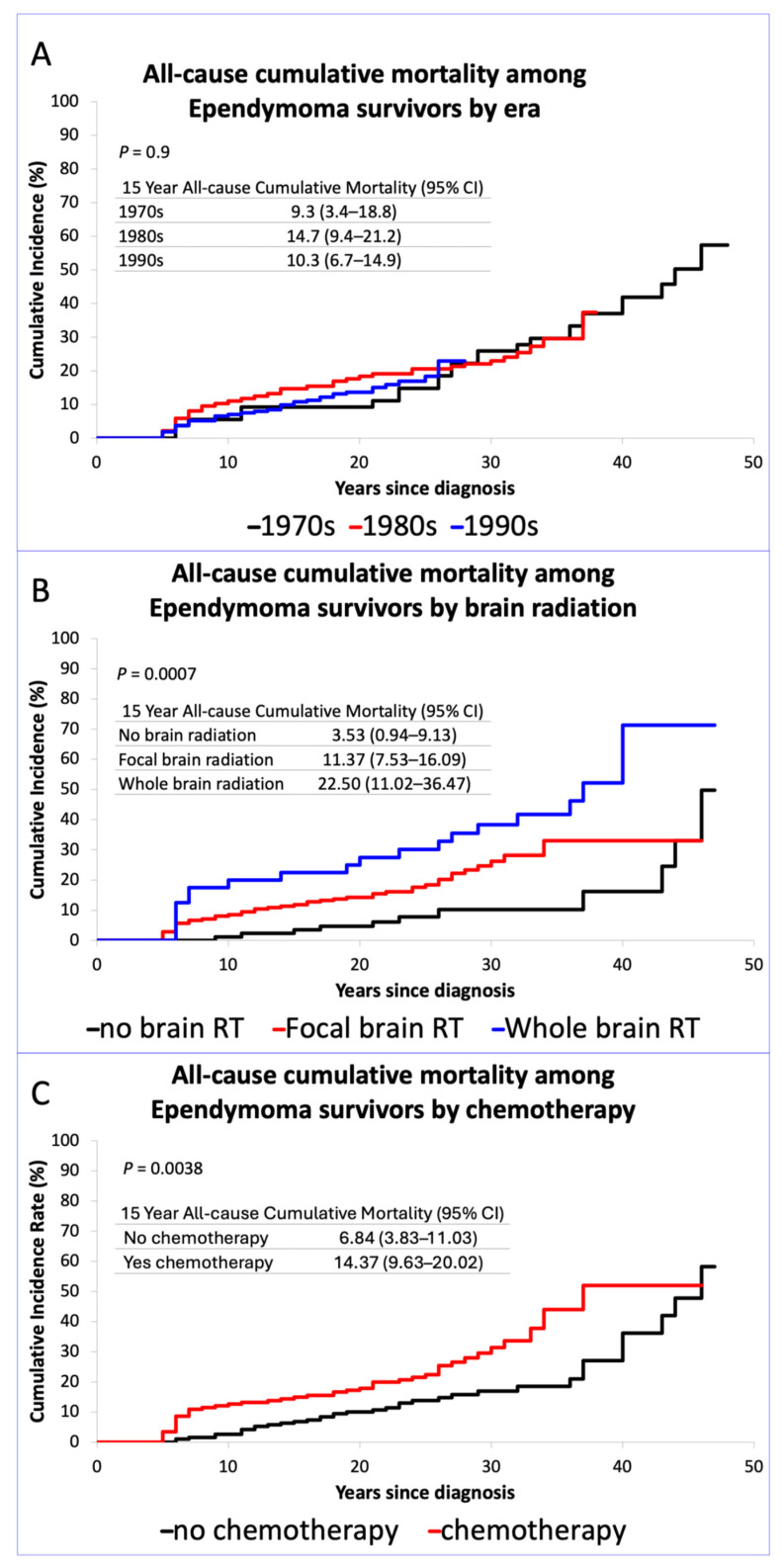
(**A**) Cumulative incidence curve for all-cause mortality by era, Inset table shows cumulative incidence of mortality at 15 years by treatment era; (**B**) Cumulative incidence curve for all-cause mortality by brain radiation treatment, Inset table shows cumulative incidence of mortality at 15 years by brain radiation treatment; (**C**) Cumulative incidence curve for all-cause mortality by chemotherapy, Inset table shows cumulative incidence of mortality at 15 years by chemotherapy.

Fifteen-year all-cause late mortality (incidence, 95% CI) estimates were similar across treatment eras: 1970s (9.3%, 3.4–18.8%), 1980s (14.7%, 9.4–21.2%), and 1990s (10.3%, 6.7–14.9%), (*p* = 0.9, comparison of cumulative incidence curves), Figure 1A. Fifteen-year all-cause late mortality by brain radiation treatment type was higher with whole-brain radiation (22.5%, 11.0–36.5%) compared to focal radiation (11.4%, 7.5–16.1%) or no brain radiation (3.5%, 0.9–9.1%), (*p* < 0.001), Figure 1B. Mortality was also higher with chemotherapy (14.4%, 9.6–20.0%) compared to no chemotherapy (6.8%, 3.8–11.0%) (*p* = 0.004), Figure 1C. Cause-specific mortality by treatment era is provided in Table S3. There were no differences in relative risk of all-cause or cause-specific late mortality by treatment era, Table 2. Relative risk of all-cause late mortality was increased with chemotherapy (RR 1.87, 1.11–3.14) (*p* = 0.02) compared to no chemotherapy, and with whole-brain radiation (3.93, 1.81–8.54) and focal brain radiation (2.25, 1.11–4.58) compared to no brain radiation (*p* = 0.002), Table 3.

Because perceived risk factors and early tumor recurrence may have influenced treatment exposure in the first five years from diagnosis, we attempted to adjust for characteristics that might predict more aggressive disease. When adjusted for anaplastic subtype and recurrence within 5 years, the relative risk of all-cause late mortality remained increased following chemotherapy exposure (RR 1.76, 1.02–3.01) compared to no chemotherapy, and with whole-brain radiation (3.69, 1.64–8.29) compared to no brain radiation, Table S4.

### 3.2. Chronic Health Conditions

Prevalence of CHCs by grade and by system are reported in Table S5. The relative risk of having any grade 3–4 CHCs, two or more grade 3–4 CHCs, or any hearing CHCs was not different across the three decades, Table S6. Compared to no brain radiation, there were increased risks (RR, 95% CI) of any grade 3–4 CHCs for focal (2.6, 1.3–5.4) and whole-brain radiation (3.5, 1.5–8.1), and two or more Grade 3–4 CHCs for whole-brain radiation (6.5, 1.3–31.6), Table 4. Relatively few chronic health conditions were reported for specific organ systems, making the calculation of relative risk impossible or not significant other than auditory. There was an increased risk of any grade 3–4 hearing loss with focal and whole-brain radiation. There were no differences in CHCs by chemotherapy exposure.

### 3.3. Subsequent Neoplasm

There were 42 SNs identified in the cohort, including 28 SMNs. The most common SNs were Meningioma (*n* = 13) and Basal Cell Carcinoma (*n* = 10). The median time to development of SN was 19.0 years (range 8.2–36.2). A list of the SN identified in the cohort is detailed in Table S7. Risk of SN or SMN was not different across treatment decades, Table S8. There was no difference in relative risk of any SMN between radiation treatment groups. The relative risk of any SN was 2.8 (1.0–7.7) for focal and 3.8 (1.2–12.4) for whole-brain RT (*p* = 0.07) compared to no brain radiation, Table 5. Chemotherapy did not affect the risk of SN or SMN.

## 4. Discussion

With 404 survivors followed for a median of 22 years, this manuscript describes the largest reported cohort of five-year survivors of childhood ependymoma. The major finding of this work is the lack of improvement in late morbidity and mortality in children and adolescents diagnosed and treated with ependymoma between 1970 and 1999. This is disappointing, considering the improvements in long term survival that have been documented in other pediatric malignancies over the same decades, with reductions in late mortality for survivors of acute lymphocytic leukemia, Wilms tumors and Hodgkin lymphoma treated in more recent decades [14]. Among childhood brain tumor survivors, recent reports from the CCSS of pediatric glioma and medulloblastoma survivors have demonstrated reductions in all-cause mortality and CHCs by nearly half over this period [15,16], but similar improvements were not observed among pediatric ependymoma survivors. Improvements for other pediatric malignancies have been attributed to changes in therapy, including a reduction in radiation exposure. Therapy for pediatric ependymoma during this time also evolved to reduce radiation exposure from whole-brain to focal therapy and increase exposure to chemotherapy. The latter possibly contributed to a lack of reduction in overall mortality. Our study shows a reduction in risk of late mortality, severe or life-threatening (grade 3–4) CHCs, and SNs among survivors of pediatric ependymoma who were exposed to reduced cranial radiation, similar to other studies. However, despite a reduction in cranial radiation exposure over time, survivors of pediatric ependymoma did not demonstrate improvements in late morbidity or mortality across the three eras.

Unlike radiation, the role of chemotherapy in ependymoma therapy is less certain. Decades of chemotherapy trials have investigated multiple chemotherapy regimens in an effort to improve outcomes. While some patients had objective tumor responses, there was no survival benefit [7]. The most recent phase III Children’s Oncology Group trial of newly diagnosed pediatric ependymoma added four cycles of maintenance chemotherapy to radiation in patients who had undergone a gross total or near total resection but survival statistics are not yet available [9]. The current International Society of Pediatric Oncology (SIOP) ependymoma study also investigates whether the addition of chemotherapy improves outcomes [24]. Neither study was stratified upfront by ependymoma subgroups based on molecular differences, and it is unknown whether a specific subgroup may benefit from chemotherapy [25].

The effect of chemotherapy exposure, as well as cranial radiation, in our study may be subject to confounding by indication, for which we cannot fully adjust. Survivors with high-risk features, such as young age or anaplastic subtype, may have been exposed to expanded therapy because of their perceived risk factors. Survivors with early recurrence (prior to cohort entry) may have been treated with additional therapy following relapse. Although adjustment of mortality analyses for these factors demonstrated no significant change in the unadjusted estimates, we were unable to adjust for all risk factors, such as tumor location and the extent of resection, which are not captured in the CCSS. We identified few differences in risk factors over the treatment decades. While survivors were younger at diagnosis in the 1980s and 1990s, other risk factors such as anaplastic histology and early tumor recurrence were not different. Survivors exposed to whole-brain radiation also did not appear to have increased risk factors compared to those exposed to focal radiation: they were older at diagnosis, tended toward less frequent anaplastic histology, and showed no difference in early tumor recurrence. On the other hand, survivors exposed to chemotherapy were associated with increased risk factors: they were younger at diagnosis, more likely to have anaplastic histology, and more frequently had early tumor recurrence. It seems reasonable that some of the worse outcomes associated with chemotherapy may be due to more aggressive disease in this treatment group. A prospective study that controls for these risk factors would be required to confirm these findings associated with chemotherapy exposure.

Regardless of the cause, the lack of improvement in late mortality over three decades is concerning. In addition to treatment changes, there are other improvements in care during this period. The incorporation of MRI into clinical care in the 1980s, improved understanding of the importance of complete resection, and improved survivorship care would be expected to improve mortality from the 1970s to 2000. The Central Brain Tumor Registry of the United States reported improvement in the overall 5-year survival of patients diagnosed with ependymoma from 1977 (29.5%) to 2011 (71.1%). The trend towards improved overall 5-year survival would have been expected to correlate with improved late mortality. The persistent late mortality, despite these advancements, highlights a critical gap in translating improved treatments and survivorship care into long-term survival benefits for pediatric ependymoma survivors. Knowledge about potential late complications should inform therapeutic strategies for patients with ependymoma going forward. Focal compared to whole-brain should be prioritized when appropriate and feasible. Survivorship care should focus on early screening for CHCs known to impact survivors. And, given the auditory impact of ependymoma therapy, investigation of otoprotectants should be prioritized in future protocols.

This study confirms the poor late outcomes found in smaller cohorts with a shorter follow-up time. Late mortality among survivors of childhood ependymoma diagnosed in the 1970s is greater than 50% 45 years from diagnosis, Figure 1A. Cranial radiation, especially whole-brain radiation, is associated with an increased risk of severe or life-threatening CHCs, as well as an increased risk of SN. These findings are similar to a recent population-based study from Canada evaluating 96 survivors of pediatric ependymoma diagnosed between 1987 and 2015 that showed an increased risk of death and disability compared to matched population controls [11]. Our study also agrees with prior studies demonstrating the significant risk of late mortality among survivors of pediatric ependymoma [26,27]. However, our study is the first to identify the lack of improvement for survivors of pediatric ependymoma over three decades, as well as identify potential late risks of chemotherapy exposure.

This study is subject to potential limitations. Chronic medical conditions in the CCSS are self-reported. This concern is partially mitigated by restricting the analysis to severe outcomes such as grade 3–4 CHCs. In addition, the years 1970–1999 saw considerable advances in diagnostic imaging and surgical techniques that may impact tumor resection, and therefore late mortality and CHCs. The extent of surgical resection is not captured in the CCSS. The concern for variable rates of gross total resection is partially addressed by reports that rates of gross total resection did not change significantly until the late 1990s. The relative stability of these rates over the decades studied may mitigate the effect this information could have added to this analysis [4,28,29,30,31]. It is likely that the majority of long-term survivors did undergo gross total resection, as survival rates for subtotal resection during this time period were poor. Almost a quarter of ependymoma patients in this cohort did not receive brain radiation. A proportion of these patients (6%) received isolated spinal radiation, which was not included in this analysis. Like other cohorts of pediatric ependymoma [11], these tumors may have been treated with surgery alone which has been shown to successfully treat some cases of pediatric ependymoma [2]. Information on molecular classification of tumors is also unavailable, although the full impact of this classification on risk stratification is still unknown. While there were changes in therapy over the decades studied, the initiation of a large cooperative trial in North America for ependymoma patients, ACNS0121, and the publication by Merchant et al. detailing the benefits of focal radiation both occurred after [3,32]. Studying these decades may have been too early to demonstrate a difference as another, albeit smaller, cohort studied through 2014 showed survival improvement [33].

## 5. Conclusions

Our study focuses on outcomes occurring five or more years after diagnosis. While prior studies have shown improved survival in pediatric ependymoma, these gains are largely limited to the first five years post-diagnosis [1,26]. In contrast, late morbidity and mortality are increasingly driven by CHCs and SNs, in addition to the late relapse that is particularly seen in ependymoma. Other CCSS studies have demonstrated long-term improvements in outcomes across cancer types, attributed to evolving therapies, technological advances and enhanced survivorship care. However, despite treatment changes in pediatric ependymoma therapy between the 1970s and 1990s, late outcomes remained unchanged. Reductions in cranial radiation treatment volumes have reduced risks of late mortality, CHC, and SN. In contrast, the introduction of chemotherapy was associated with increased risks of late mortality, although these results should be interpreted with caution. Further research is needed to understand this lack of progress in late outcomes and to evaluate the long-term risks of emerging therapies in future trials.

For oncologists, these results underscore the need for long-term surveillance and multidisciplinary survivorship care that extends well beyond the traditional five-year window, as adult survivors of childhood ependymoma remain at risk for late mortality, chronic health conditions, and subsequent neoplasms related to prior treatments. Importantly, this study demonstrates that focusing only on early survival endpoints fails to capture the true burden of disease. Given that approximately 70% of patients with pediatric ependymoma survive at least five years but remain at risk for recurrence and late morbidity, future clinical trials should incorporate longer observation periods to more accurately assess the lasting risks and benefits of novel therapies. Despite therapeutic advances, expected improvements in late outcomes have not yet been realized, underscoring the need for continued research and comprehensive survivorship strategies to improve long-term quality of life.

## Figures and Tables

**Table 1:** Demographic and treatment characteristics for five-year survivors of childhood ependymoma: overall and by treatment era.

	Total	1970–1979	1980–1989	1990–1999	
Characteristic	*n* = 404	*n* = 55	*n* = 136	*n* = 213	*p*-Value *^a^*
**Diagnosis**					
9383.1—subependymoma	4 (0.99)	1 (1.8)	1 (0.7)	2 (0.9)	0.6
9391.3—ependymoma NOS	342 (84.65)	48 (87.3)	118 (86.8)	176 (82.6)	
9392.3—anaplastic ependymoma,	36 (8.91)	2 (3.6)	10 (7.4)	24 (11.3)	
9393.1—papillary ependymoma	6 (1.49)	2 (3.6)	1 (0.7)	3 (1.4)	
9394.1—myxopapillary ependymoma	16 (3.96)	2 (3.6)	6 (4.4)	8 (3.8)	
**Sex (*n*,%):** Male	212 (52.5)	32 (58.2)	65 (47.8)	115 (54.0)	0.35
Female	192 (47.5)	23 (41.8)	71 (52.2)	98 (46.0)	
**Race/Ethnicity (*n*,%):** Non-Hispanic White	326 (80.7)	50 (90.9)	117 (86.0)	159 (74.6)	**0.006**
Non-Hispanic Black	30 (7.4)	0 (0.0)	6 (4.4)	24 (11.3)	
Hispanic	33 (8.2)	2 (3.6)	8 (5.9)	23 (10.8)	
Other	15 (3.7)	3 (5.5)	5 (3.7)	7 (3.3)	
**Age at diagnosis (median, min–max) years**	6 (0–20)	10 (0–19)	6 (0–20)	5 (0–20)	**0.0001**
**Time since diagnosis (median, min–max) years**	22 (5–49)	34 (6–49)	27.5 (5–37)	20 (5–28)	**<0.001**
**Chemotherapy Exposure (*n*,%)**	174 (47.7)	13 (29.5)	59 (50)	102 (50.2)	**0.037**
**Any surgery (*n*,%)**	367 (100)	45 (100)	118 (100)	204 (100)	NA *^b^*
**Any brain RT (*n*,%)**	265 (75.7)	28 (65.1)	97 (84.3)	140 (72.9)	**0.0174**
**Radiation treatment group, dose ≥40 Gy**					
Dose in Gy, among those ≥40 Gy (median, min–max)	54 (40–300)	50 (40–69)	54 (43.2–90)	56 (40–300)	**<0.0001**
No brain radiation	85 (25.2)	15 (35.7)	18 (16.1)	52 (28.4)	**<0.0001**
Focal brain radiation	212 (62.9)	9 (21.4)	77 (68.8)	126 (68.9)	
Whole-brain radiation	40 (11.9)	18 (42.9)	17 (15.2)	5 (2.7)	
**Recurrence within 5 years (*n*,%)**	48 (11.9)	3 (5.5)	16 (11.8)	29 (13.6)	0.25
**Age at radiation in years (median, min–max)**	5.45 (0.8–21.35)	9.33 (1.19–16.96)	5.39 (0.8–20.96)	5.41 (0.92–21.35)	0.1815

*^a^* Overall *p* value. *^b^* NA—All patients with known treatment information had surgery, therefore *p*-value cannot be calculated. The percentages were based on the total number of participants for whom information was available.

**Table 2:** Relative risk of late mortality among survivors of ependymoma, adjusted for treatment decade *^a^*.

	All-Cause Mortality	Death due to Recurrence or Progression of Primary Malignancy	Health-Related Cause Mortality
	RR [95%CI]	RR [95%CI]	RR [95%CI]
**Treatment Era**			
1970–1979	1.0	1.0	1.0
1980–1989	0.95 (0.54–1.69)	1.74 (0.54–5.64)	0.68 (0.31–1.5)
1990–1999	0.88 (0.47–1.65)	1.51 (0.46–4.99)	0.85 (0.35–2.09)
**Sex**			
Male	1.0	1.0	1.0
Female	0.95 (0.63–1.41)	1.21 (0.64–2.28)	0.84 (0.46–1.51)
**Race**			
Non-Hispanic White	1.0	1.0	1.0
Other	0.92 (0.52–1.61)	0.93 (0.4–2.13)	0.85 (0.35–2.04)

*^a^* Multivariable models adjusted for attained age.

**Table 3:** Relative risk of late mortality among survivors of ependymoma, adjusted for chemotherapy and brain radiation *^a^*.

	All-Cause	Recurrence or Progression of Primary Malignancy	Health-Related Causes
	RR [95%CI]	RR [95%CI]	RR [95%CI]
**Sex**			
Male	1.0	1.0	1.0
female	0.92 (0.58–1.46)	1.24 (0.58–2.67)	0.78 (0.39–1.55)
**Race**			
Non-Hispanic White	1.0	1.0	1.0
Other	0.71 (0.36–1.4)	1.13 (0.45–2.83)	0.29 (0.07–1.21)
**Chemotherapy**			
No	1.0	1.0	1.0
Yes	**1.87 (1.11–3.14)**	1.56 (0.68–3.58)	**2.93 (1.31–6.54)**
**Brain Radiation**			
No brain radiation	1.0	1.0	1.0
Focal brain radiation	**2.25 (1.11–4.58)**	3.98 (0.92–17.33)	1.44 (0.54–3.85)
Whole-brain radiation	**3.93 (1.81–8.54)**	5.2 (0.94–28.6)	**3.86 (1.43–10.43)**

*^a^* Multivariable models adjusted for attained age.

**Table 4:** Relative risk of chronic health conditions among survivors of ependymoma, adjusted for chemotherapy and radiation *^a^*.

	Any Grade 3–4	>1 Grade 3–4	Any Hearing CHC Grade 3–4
	RR [95%CI]	RR [95%CI]	RR [95%CI]
**Sex**			
Male	1.0	1.0	1.0
Female	1.13 (0.69–1.84)	1.09 (0.52–2.27)	0.43 (0.18–1.06)
**Race**			
Non-Hispanic White	1.0	1.0	1.0
Other	1.13 (0.61–2.1)	1.42 (0.60–3.37)	0.56 (0.17–1.90)
**Chemotherapy**			
No	1.0	1.0	1.0
Yes	1.15 (0.69–1.91)	2.17 (0.99–4.73)	0.55 (0.22–1.33)
**Brain Radiation**			
No Brain Radiation	1.0	1.0	1.0
Focal Brain Radiation	**2.6 (1.25–5.39)**	4.39 (1.0–19.31)	**12.54 (1.60–98.14)**
Whole-Brain Radiation	**3.5 (1.48–8.11)**	**6.47 (1.32–31.64)**	**15.65 (1.79–137.07)**

*^a^* Multivariable models adjusted for attained age.

**Table 5:** Relative risk of subsequent neoplasm in five-year survivors of childhood ependymoma, adjusted for treatment exposure *^a^*.

	Any Subsequent Neoplasm	Any Subsequent Malignant Neoplasm
	RR [95%CI]	RR [95%CI]
**Sex**		
Male	1.0	1.0
Female	1.73 (0.86–3.49)	1.16 (0.49–2.75)
**Race**		
Non-Hispanic White	1.0	1.0
Other	0.45 (0.14–1.47)	0.48 (0.11–2.08)
**Chemotherapy**		
No	1.0	1.0
Yes	1.26 (0.62–2.58)	1.67 (0.68–4.11)
**RT**		
No Brain Radiation	1.0	1.0
Focal Brain Radiation	2.78 (1.0–7.73)	2.70 (0.74–9.93)
Whole-Brain Radiation	**3.82 (1.17–12.41)**	3.05 (0.66–13.98)

*^a^* Multivariable models adjusted for attained age.

## Data Availability

The Childhood Cancer Survivor Study is a US National Cancer Institute funded resource (U24 CA55727) to promote and facilitate research among long-term survivors of cancer diagnosed during childhood and adolescence. CCSS data are publicly available on dbGaP at https://www.ncbi.nlm.nih.gov/gap/ (accessed on 9 November 2025) through its accession number phs001327.v2.p1., and on the St Jude Survivorship Portal within the St. Jude Cloud, at https://survivorship.stjude.cloud/ (accessed on 9 November 2025). In addition, utilization of the CCSS data that leverages the expertise of CCSS Statistical and Survivorship research and resources will be considered on a case-by case basis. For this utilization, a research application of intent, followed by an analysis concept proposal, must be submitted for evaluation by the CCSS Publications Committee. Users interested in utilizing this resource are encouraged to visit http://ccss.stjude.org (accessed on 9 November 2025). Full analytical data sets associated with CCSS publications since January of 2023 are also available on the St. Jude Survivorship Portal at https://viz.stjude.cloud/community/cancer-survivorship-community~4/publications (accessed on 9 November 2025).

## Supplementary Materials

The following supporting information can be downloaded at: https://www.mdpi.com/article/10.3390/cancers17223669/s1. Table S1: Chemotherapy agents; Table S2: Characteristics of ependymoma survivors by treatment type; Table S3: Cause-specific late mortality by era; Table S4: Relative risk of late mortality among survivors of ependymoma, adjusted for treatment, recurrence within 5 years, and ependymoma subtype (9391.3 and 9392.3) *^a^*; Table S5: Prevalence of chronic health conditions in five-year survivors of ependymoma by decade; Table S6: Relative risk of chronic health conditions in five-year survivors of ependymoma adjusted for treatment decade *^a^*; Table S7: Subsequent neoplasms among patients with known treatment information; Table S8: Multivariable relative risk of subsequent neoplasm by treatment era *^a^.*

## Abbreviations

The following abbreviations are used in this manuscript:

CCSS	Childhood Cancer Survivor Study
NCI	National Cancer Institute
ICD-9 and ICD-10	International Classification of Diseases, Ninth and Tenth Revisions
CHCs	Chronic Health Conditions
CTCAE	Common Terminology Criteria for Adverse Events
SNs	Subsequent Neoplasms
SMNs	Subsequent Malignant Neoplasms
ICD-O, 3rd version	International Classification of Diseases for Oncology
RR	Relative Risk
SIOP	International Society of Pediatric Oncology
