# Supplementary material for: Late Morbidity and Mortality in Survivors of Childhood Ependymoma: A Report from the Childhood Cancer Survivor Study (CCSS)
Source: Cancers (Basel). 2025 Nov 15;17(22):3669. doi: 10.3390/cancers17223669 (PMC12650955; doi:10.3390/cancers17223669)
Supplement: Supplementary file 1 [file cancers-17-03669-s001.zip › cancers-3925950-supplementary.pdf]

cancers

MDPI

Supplementary Materials: Late Morbidity and Mortality in Survivors of Childhood Ependymoma: A Report from the Childhood Cancer Survivor Study (CCSS)

Katharine R. Lange, Peter de Blank, Mengqi Xing, Sedigheh Mirzaei, Deo Kumar Srivastava, Kevin Oeffinger, Joseph Neglia, Kevin Krull, Paul C. Nathan, Rebecca Howell, Kirsten K. Ness, Lucie M. Turcotte, Wendy Leisenring, Gregory T. Armstrong, Tara Brinkman, Daniel C. Bowers and Mehmet Fatih Okcu

Table S1. Chemotherapy Agents.

Chemotherapy drug	1970–1979		1980–1989		1990–1999	
	N[a]	%[b]	N[a]	%[b]	N[a]	%[b]
BCNU (Carmustine)	0	0	5	1.86	0	0
CCNU (Lomustine)	9	28.13	34	12.64	5	1.34
Carboplatin	0	0	6	2.23	46	12.3
Cis-Platinum	1	3.13	34	12.64	48	12.83
Cyclophosphamide (Cytoxan)	2	6.25	28	10.41	61	16.31
Cytosine Arabinoside (Ara-C)	0	0	20	7.43	2	0.53
Dexamethasone	1	3.13	9	3.35	10	2.67
Doxorubicin (Adriamycin)	0	0	1	0.37	1	0.27
DTIC	0	0	1	0.37	0	0
Hydroxyurea (Hydrea)	0	0	13	4.83	2	0.53
Idarubicin	0	0	0	0	1	0.27
Ifosfamide	0	0	2	0.74	21	5.61
6-Mercaptopurine (6 MP)	0	0	1	0.37	0	0
Melphalan	0	0	0	0	1	0.27
Methotrexate	1	3.13	6	2.23	1	0.27
Nitrogen Mustard	0	0	6	2.23	1	0.27
Prednisone	6	18.75	20	7.43	3	0.8
Procarbazine	2	6.25	28	10.41	4	1.07
6-Thioguanine (6 TG)	0	0	1	0.37	2	0.53
Thiotepa	0	0	2	0.74	4	1.07
Vincristine	10	31.25	43	15.99	77	20.59
VP-16 (Etoposide)	0	0	9	3.35	81	21.66
Irinotecan (Camptosar)	0	0	0	0	1	0.27
Temozolomide (Temodar)	0	0	0	0	2	0.53

[a]N is number of patients who received each drug. [b]% is percentage of exposure to an individual drug among all exposures in the decade.

 https://doi.org/10.3390/cancers17223669

Table S2. Characteristics of Ependymoma Survivors by Treatment Type.

		Brain Radiation					Chemotherapy		
		No brain Radiation	Focal brain Radiation	Whole brain Radiation	*p*-value[a]	*p*-value[b]	No Chemo	Chemo	*p*-value
**Age at Diagnosis (median, min-max) years**		9 (0–20)	4 (0–20)	7.5 (1–19)	**0.0001**	**0.017**	9 (0–20)	3 (0–20)	**<0.0001**
**Treatment with Chemotherapy**	No	61 (72.6)	94 (44.5)	18 (50.0)	**<0.0001**	0.54	NA	NA	NA
	Yes	23 (27.4)	117 (55.5)	18 (50.0)			NA	NA	
**Treatment with Brain Radiation**	No	NA	NA	NA	NA	NA	61 (33.7)	23 (14.1)	**<0.0001**
	Yes	NA	NA	NA	NA	NA	120 (66.3)	140 (85.9)	
**Anaplastic Ependymoma Subtype (percent among all ependymoma subtypes)**	Yes	0 (0.0)	31 (14.6)	2 (5.0)	**<0.0001**	0.094	8 (4.2)	26 (14.9)	**<0.0001**
**Recurrence within 5 years (N,%)**	No	80 (94.1)	185 (87.3)	33 (82.5)	0.11	0.42	174 (91.1)	147 (84.5)	0.05
	Yes	5 (5.9)	27 (12.7)	7 (17.5)			17 (8.9)	27 (15.5)	

[a]Overall p-value comparing three groups. [b]Overall p-value comparing focal vs whole brain radiation.

Table S3. Cause-Specific Late Mortality by Era.

	1970–1979	1980–1989	1990–1999
**Cause-specific mortality, %[a] among dead population**	**N = 23**	**N = 30**	**N = 34**
Recurrence or progression of primary childhood ependymoma	4 (17.4)	16 (53.33)	19 (55.9)
External cause	2 (8.7)	0 (0.0)	1 (2.94)
Subsequent Neoplasm and Health-related cause	17 (73.91)	14 (46.7)	14 (41.2)

[a]The percentages were based on the total number of participants for whom information was available.

Table S4. Relative Risk of late mortality among survivors of Ependymoma, adjusted for treatment, recurrence within 5 years, and ependymoma subtype (9391.3 and 9392.3)[a].

	**All-Cause**	**Recurrence or Progression of Primary Malignancy**	**Health-Related Causes**
	RR [95%CI]	RR [95%CI]	RR [95%CI]
**Sex**			
Male	1.0	1.0	1.0
female	0.9 (0.55–1.45)	1.2 (0.55–2.64)	0.76 (0.37–1.57)
**Race**			
Non-Hispanic white	1.0	1.0	1.0
Other	0.92 (0.46–1.82)	1.45 (0.57–3.73)	0.35 (0.08–1.52)
**Chemotherapy**			
No	1.0	1.0	1.0
Yes	**1.76 (1.02–3.01)**	1.37 (0.58–3.25)	**3.16 (1.34–7.47)**
**Brain Radiation**			
No brain radiation	1.0	1.0	1.0
Focal brain radiation	1.80 (0.84–3.88)	2.595 (0.578–11.660)	1.309 (0.446–3.845)
Whole brain radiation	**3.69 (1.64–8.29)**	4.22 (0.77–23.29)	**3.95 (1.35–11.52)**
**Recurrence within 5 years**			
No	1.0	1.0	1.0
Yes	**6.1 (3.44–10.81)**	**5.78 (2.48–13.48)**	**5.92 (2.23–15.70)**
**Ependymoma subtype**			
9391.3 – ependymoma NOS	1.0	1.0	1.0
9392.3 – anaplastic ependymoma	1.27 (0.59–2.75)	1.49 (0.48–4.56)	0.97 (0.22–4.3)

[a]Multivariable models adjusted for attained age .

Table S5. Prevalence of Chronic Health Conditions in Five-year Survivors of Ependymoma by decade.

	Total	1970-1979	1980-1989	1990-1999
CHC conditions (N,%)	N=404	N=55	N=136	N=213
Any grade 1-4	253 (62.6)	42 (76.4)	94 (69.1)	117 (54.9)
Any grade 3-4	101 (25.0)	18 (32.7)	47 (34.6)	36 (16.9)
>1 Grade 3-4	41 (10.1)	7 (12.7)	19 (14.0)	15 (7.0)
Any Endocrine CHC grade 3-4	22 (5.4)	4 (7.3)	14 (10.3)	4 (1.9)
Any Cardiac CHC grade 3-4	17 (4.2)	4 (7.3)	6 (4.4)	7 (3.3)
Any Pulm CHC grade 3-4	1 (0.2)	0 (0.0)	1 (0.7)	0 (0.0)
Any Neuro CHC grade 3-4	39 (9.7)	8 (14.5)	18 (13.2)	13 (6.1)
Any Vision CHC grade 3-4	11 (2.7)	4 (7.3)	4 (2.9)	3 (1.4)
Any Hearing CHC grade 3-4	36 (8.9)	5 (9.1)	12 (8.8)	19 (8.9)
Any Speech CHC grade 3-4	0 (0.0)	0 (0.0)	0 (0.0)	0 (0.0)

Table S6. Relative Risk of Chronic Health Conditions in Five-year Survivors of Ependymoma adjusted for treatment decade[a].

	**Any Grade 3–4**	**>1 Grade 3–4**	**Any Hearing CHC Grade 3–4**
	RR [95%CI]	RR [95%CI]	RR [95%CI]
**Year of diagnosis**			
1970–1979	1.0	1.0	1.0
1980–1989	1.36 (0.75–2.45)	1.32 (0.54–3.25)	1.27 (0.4–4.05)
1990–1999	0.82 (0.43–1.56)	0.86 (0.32–2.28)	2.06 (0.64–6.61)

[a]Multivariable models adjusted for attained age, race, ethnicity, and sex.

Table S7. Subsequent Neoplasms among patients with known treatment information.

**Subsequent Neoplasm**	**Frequency**
Meningioma	13
Basal Cell Carcinoma	10
Papillary Thyroid Carcinoma	4
Sarcoma	3
Adenocarcinoma	2
Melanoma	2
Pilocytic Astrocytoma	2
Renal Cell Carcinoma	1
Squamous Cell Carcinoma	1
Glioblastoma	1
Mucoepidermoid Carcinoma of the Parotid Gland	1
Malignant Fibrous Histiocytoma	1
Other Malignant Neoplasm of the Brain	1

Table S8. Multivariable Relative Risk of Subsequent Neoplasm by Treatment Era[a].

	**Any Subsequent Neoplasm**	**Any Subsequent Malignant Neoplasm**
	RR [95%CI]	RR [95%CI]
**Treatment era**		
1970–1979	1.0	1.0
1980–1989	2.06 (0.85–5.0)	2.62 (0.84–8.25)
1990–1999	1.16 (0.42–3.17)	1.55 (0.42–5.70)

[a]Multivariable models adjusted for attained age, race, ethnicity, and sex
